# The Standard, Intervention Measures and Health Risk for High Water Iodine Areas

**DOI:** 10.1371/journal.pone.0089608

**Published:** 2014-02-28

**Authors:** Peng Liu, Lixiang Liu, Hongmei Shen, Qingzhen Jia, Jinbiao Wang, Heming Zheng, Jing Ma, Dan Zhou, Shoujun Liu, Xiaohui Su

**Affiliations:** 1 Centre for Endemic Disease Control, Chinese Centre for Disease Control and Prevention, Harbin Medical University, Harbin, Heilongjiang, China; 2 Institute for Endemic Disease Control of Shanxi Province, Linfen, Shanxi, China; 3 Institute for Endemic Disease Control of Shandong Province, Jinan, Shandong, China; 4 Centre for Disease Control of Henan Province, Zhengzhou, Henan, China; 5 Centre for Disease Control of Hebei Province, Shijiazhuang, Hebei, China; The Ohio State University, United States of America

## Abstract

Our study aims to clarify the population nutrient status in locations with different levels of iodine in the water in China; to choose effective measurements of water improvement(finding other drinking water source of iodine not excess) or non-iodised salt supply or combinations thereof; to classify the areas of elevated water iodine levels and the areas with endemic goiter; and to evaluate the risk factors of water iodine excess on pregnant women, lactating women and the overall population of women. From Henan, Hebei, Shandong and Shanxi province of China, for each of 50∼99 µg/L, 100∼149 µg/L, 150∼299 µg/L, and ≥300 µg/L water iodine level, three villages were selected respectively. Students of 6–12 years old and pregnant were sampled from villages of each water-iodine level of each province, excluded iodized salt consumer. Then the children's goiter volume, the children and pregnant's urinary iodine and water iodine were tested. In addition, blood samples were collected from pregnant women, lactating women and other women of reproductive age for each water iodine level in the Shanxi Province for thyroid function tests. These indicators should be matched for each person. When the water iodine exceeds 100 µg/L; the iodine nutrient of children are iodine excessive, and are adequate or more than adequate for the pregnant women. It is reasonable to define elevated water iodine areas as locations where the water iodine levels exceed 100 µg/L. The supply of non-iodised salt alone cannot ensure adequate iodine nutrition of the residents, and water improvement must be adopted, as well. Iodine excess increases the risk of certain thyroid diseases in women from one- to eightfold.

## Introduction

Iodine excess can be caused by various sources. In 1960, Suzuki reported cases of goiter in Hokkaido caused by eating kelp[1],[2], and Mecullagh observed that the goiter prevalence increase after iodised oil injection[3]. In 1978[4] and 1987[5], China reported the high iodine goitres caused by water iodine. In 2013, there were ten countries with excessive iodine intake[6] secondary to numerous factors. Currently, although iodine excess caused by elevated water iodine has been reported mainly in China, some other countries also suffer this problem, such as Columbia[7], Sudan[8], Germany[9], Denmark[10], Niger[11], Saharawi[12] and Sri Lanka[13]. In China, water iodine levels vary greatly amongst the elevated water iodine areas spread across 12 provinces and municipalities[14]. However, the supply of non-iodised salt has been adopted for these areas as the only intervention measure, with the exception of Beijing and Fujian, where water improvement has also been used. Hence, several questions remain: 1) For different areas with elevated water iodine levels, what are the iodine levels in the population? 2) Should water improvement, the supply of non-iodised salt, or a combination of both interventions be adopted based on the specific context of relevant areas? 3) How should the areas with elevated water iodine and the areas with endemic iodine excess goitres be determined and classified? Therefore, the Study Project on the Standard and Intervention Measures for Areas of Elevated Water Iodine was implemented by the Endemic Disease Centre of China in the elevated water iodine provinces, including Shandong, Shanxi, Henan and Hebei Provinces, from July 2011 to March 2012. The project results are now shared as follows.

## Materials and Methods

### Survey scope

The number of surveyed villages was determined according to the historic IDD surveillance results in recent years and in reference to the various local water iodine levels of the four provinces. For each range of iodine levels (50–99 µg/L, 100–149 µg/L, 150–299 µg/L, and ≥300 µg/L), three villages were selected in each of the four provinces.

### Survey subjects

Children aged six to 12 years-old were sampled in the primary schools of the survey villages (should no primary schools exist in the surveyed villages, students from the village studying at the township central primary school were sampled). For each pre-set water iodine group, at least 200 children taking non-iodised salt were sampled. Pregnant women of the survey villages were sampled from the maternal and child care service centres or health centres of the townships. For each pre-set water iodine group, at least 50 pregnant women taking non-iodised salt were sampled.

### Survey indicators

Water iodine levels were verified at the surveyed villages with five water samples collected from the east, west, south, north and centre of each village with scattered water supplies, and two water samples were collected from villages with centralised water supplies, before the following survey was conducted. If the water iodine of the village fit the pre-set level, the drinking water of the children's and pregnant women's households was sampled to test the actual water iodine level. The table salt of the children's and pregnant women's households was sampled for testing through a semi-quantitative method (<5 mg/kg was defined non-iodised salt). Children and pregnant women from the households found to use iodised salt were excluded, and the remaining individuals continued with the following tests and examinations. One spot urine samples were collected from the above-mentioned children and pregnant women to test their urinary iodine levels. The urinary iodine levels were stratified according to the WHO's recommendation [15]. Goiter size was detected by ultrasound among the children aged six to 12 years-old, and goiter was defined by the Chinese diagnostic criteria for endemic goiter (WS276-2007)[16]. Approximately blood samples were collected from pregnant women, lactating women and other women of reproductive age for each water iodine level in Shanxi Province for FT3, FT4, TSH, anti-TPO and anti-Tg tests. The reference values were FT3: 2.8–7.1 pmol/L, FT4: 12–22 pmol/L, TSH: 0.27–4.20 IU/L, TPOAb: >34 IU/ml, TgAb: >115 IU/ml. The urinary iodine levels, table salt iodine, drinking water iodine (and children's goiter size or pregnant women's thyroid function) was matched for each person.

### Quality control

A detailed project implementation plan was developed and validated by experts to control the project quality. A project launching/training meeting and a review meeting were conducted by the Endemic Disease Centre, which the project managers from the project provinces, prefectures and counties attended. A centralised database was developed, and the submitted data were cleaned by the Endemic Disease Centre to ensure data quality. Project managers and relevant staff from the Endemic Disease Centre participated in the onsite work in the Shandong and Hebei Provinces. All laboratories responsible for the testing passed the external quality check by the National IDD Reference Lab.

### Ethical statement

The research was approved by the review board of Harbin Medical University. The informed consent form was prepared before the research began. We obtained informed consent from the guardians on the behalf of the children and the women themselves, and the consent was written.

## Results

### Overview of project sites

**Figure 1:**
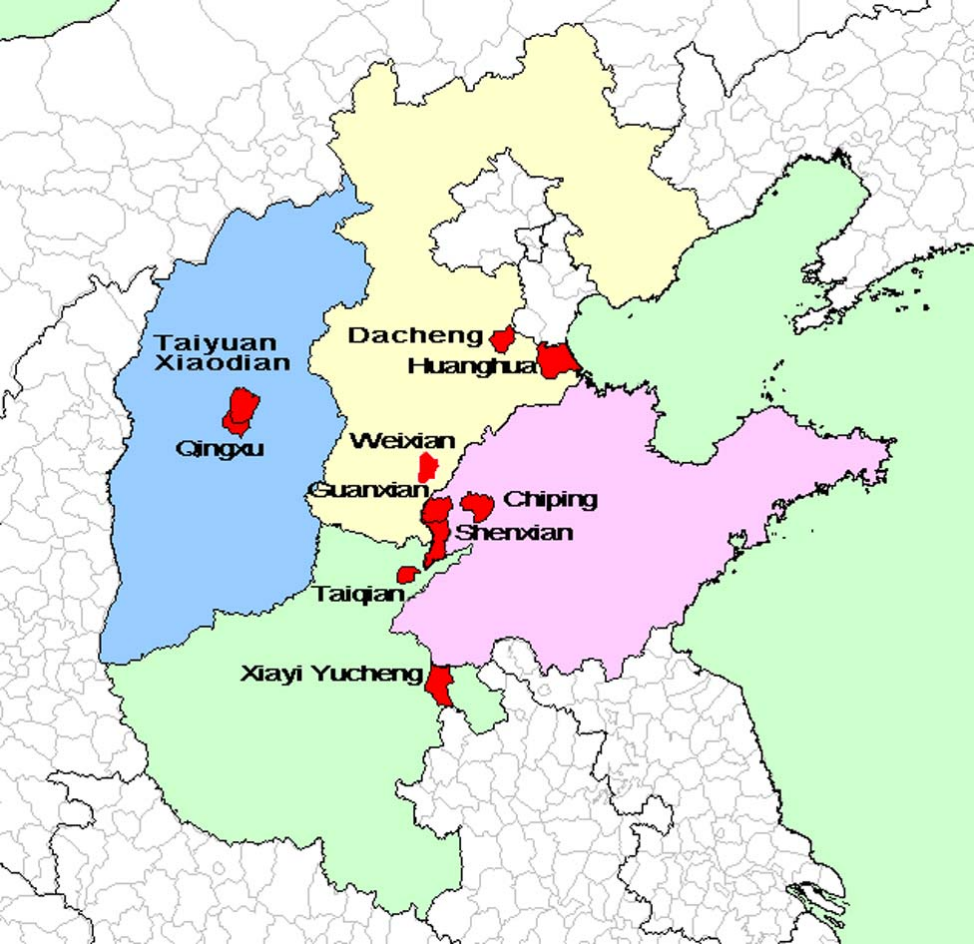
Distribution of the survey sites.

The survey counties for Shandong were Xinxian, Guanxian and Chiping in Liaocheng City; those for Shanxi were Xiaodian and Qingxu in Taiyuan City; those for Henan were Xiayi and Yucheng in Shangqiu City and Taiqian in Puyang City; and those for Hebei were Dacheng in Langfang City, Huanghua in Cangzhou City, and Weixian in Xingtai City. Please see Figure 1, Table S1 in File S1 for the survey sites.

### Profile of the survey subjects

Onsite work was then developed according to the implementation plan at the project sites. The total sample size met the size required by the implementation plan. Please see Table S2–S4 in File S1. Survey subjects were then further grouped based on the actual drinking water iodine levels. The sample size grouped by the actual water iodine levels differed only slightly from the required one and met the size requirement. The survey indicated that seven- to 11-year-old children accounted for a larger proportion of the children surveyed and that those 6 and 12 years old accounted for a smaller number. Please see Table S5 in File S1. The pregnant women surveyed were predominantly 20–30 years old, and women in this age group accounted for 80% of the total survey subjects. Please see Table S6 in File S1.

### Results of the water iodine survey

The median water iodine was calculated according to the actual water iodine levels. The actual water iodine levels of the children and pregnant are consistent with the pre-set water group; hence, we only supply results from the actual water iodine levels. The water iodine levels of the children were consistent with those of the pregnant women for the same actual water iodine group. Please see Table S7 in File S1, Figure S1,S2 in File S1.

### MUI (Median of Urinary Iodine)

The MUI of the children and pregnant women in the actual water iodine groups demonstrates that with water iodine levels of 50–99 µg/L, children exhibited more than adequate iodine levels with their MUI between 200 and 299 µg/L. When the water iodine levels exceeded 100 µg/L, the children in various groups exhibited excessive iodine levels with MUI exceeding 300 µg/L. This result is consistent across the four provinces. With water iodine levels of 50–99 µg/L, the urinary iodine of the pregnant women in the Shandong, Shanxi and Henan provinces fell within 150–249 µg/L. With the water iodine levels of 100–149 µg/L, the urinary iodine levels of the pregnant women from all provinces either fell within the adequate range of 150–249 µg/L or exceeded adequate levels (250–499 µg/L). Thus, it can be concluded that with the supply of non-iodised salt and water iodine levels of 50–99 µg/L, children exhibit more than adequate iodine levels and pregnant women exhibit adequate iodine levels; with water iodine levels between 100 and 149 µg/L, children exhibit excessive iodine levels, and pregnant women either exhibit adequate or excessive iodine levels. See Table 1, S8–S12 in File S1, Figure S3–S5 in File S1 for details.

**Table 1:** Drinking water iodine and urinary iodine grouped by the actual water iodine levels.

Province	Actual water	Children (median µg/L)			Pregnant (median µg/L)		
	iodine group (µg/L)	Size	Water iodine	Urinary iodine	Size	Water iodine	Urinary iodine
Shandong	50–99	683	71.6	252.0	142	68.3	166.2
	100–149	256	113.8	336.9	85	120.2	202.9
	150–299	302	239.3	460.4	62	201.6	278.8
	≥300	215	350.3	620.2	79	405.7	452.0
Shanxi	50–99	196	73.8	274.2	21	82.0	238.6
	100–149	189	144.7	312.8	20	121.5	204.9
	150–299	159	258.5	445.6	21	258.5	373.9
	≥300	165	501.0	793.5	19	485.9	607.4
Henan	50–99	131	64.1	270.4	26	74.9	280.9
	100–149	338	139.6	336.0	147	138.1	326.0
	150–299	383	242.8	379.0	205	224.1	308.5
	≥300	310	475.7	345.0	150	475.7	349.5
Hebei	100–149	195	111.7	396.4	50	110.7	309.8
	150–299	226	236.5	316.8	65	235.9	270.4
	≥300	211	392.3	382.7	39	335.8	385.0
Total	50–99	1010	72.2	260.2	187	69.6	183.0
	100–149	978	118.2	338.2	299	121.5	284.2
	150–299	1070	242.8	401.0	353	230.2	304.0
	≥300	901	392.3	473.0	287	411.9	386.0

Note: P<0.001 when different groups of the lumped actual household water iodine are compared with a median test. This result suggests that a significant difference exists among the water iodine levels of various groups and that such difference also exists when any two groups are compared.

### Thyroid size

Our analysis of goiter size indicated that the goiter rate is not typically high, despite the fact that most of the groups surveyed were within areas with elevated water iodine levels. The goiter rate typically exceeded 5% when the water iodine exceeded 300 µg/L. The goiter rate for all water iodine levels in Shanxi was higher than that of other provinces, although the underlying cause remains to be further. Please see Figure S6–S7 in File S1.

### Results of thyroid function testing among pregnant women (percentage tested positive)

**Figure 2:**
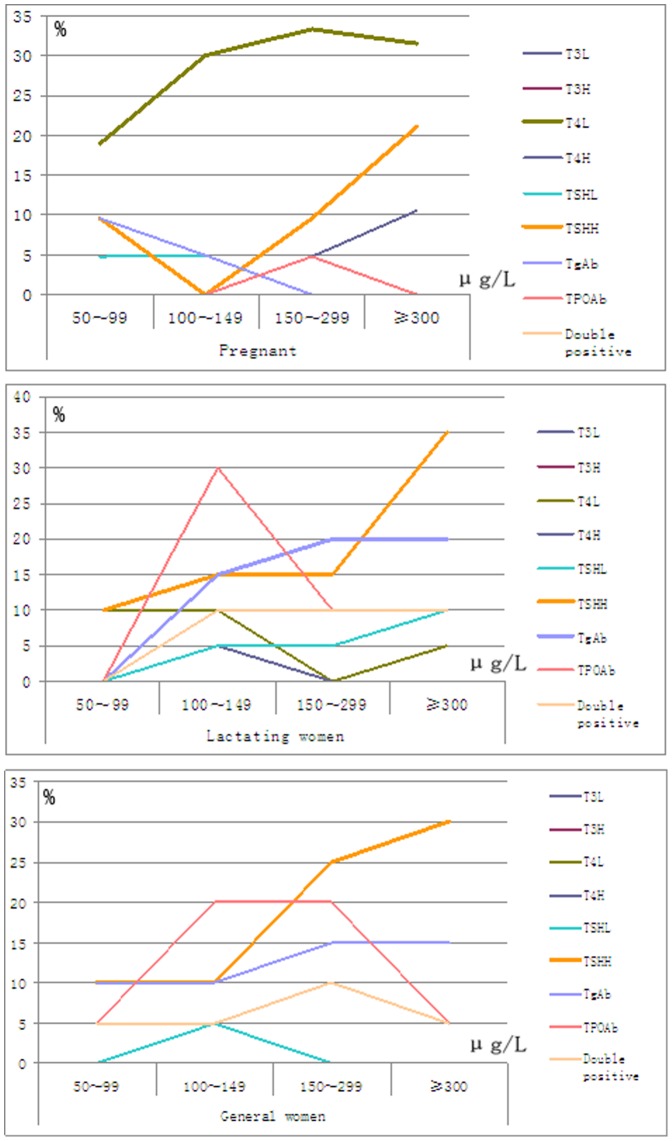
Blood tests results among pregnant (a), lactating (b) and child-bearing age woman (c).

Figure 2, Table S14 in File S1 indicates that the pregnant women with low T4 levels account for a high percentage across the groups. The proportion increases from 19.0% to 31.6% with the rising water iodine, which suggests decreasing storage of thyroid hormones. The rising water iodine levels are coupled with the rising percentage of lactating women with both low and high TSH. However, the hormone levels of the lactating women are quite stable, suggesting active physiological adjustment. In the groups of women of child-bearing age, the percentage of those with high TSH levels rises with increasing water iodine. However, their hormone levels do not register a remarkable increase, contrary to the normal situation. A possible explanation can be that the rising TSH level not only causes elevated hormone levels but also promotes the degradation of the hormones. A large number of lactating women and women of child-bearing age tested positive with either anti-TPO or anti-Tg, with the percentage reaching 30% in some groups. Furthermore, a high percentage of such women tested positive with two indicators (see Figure 2 a, b and c, Table S13, S14 in File S1).

**Figure 3:**
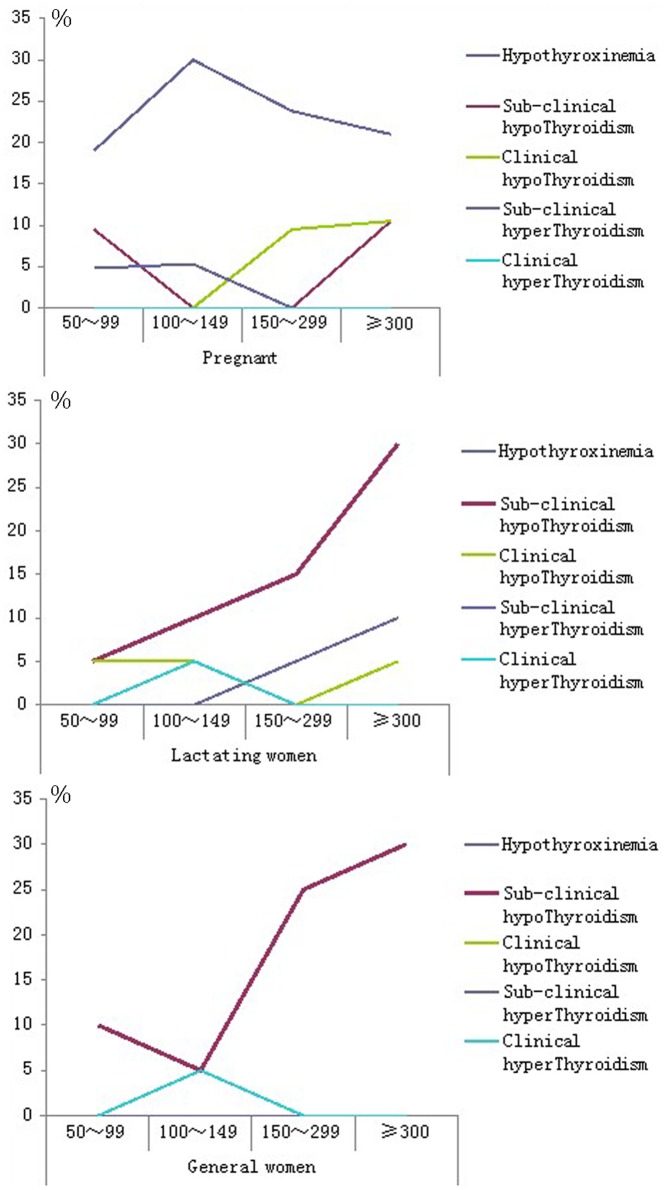
Detection rate among the pregnant (a), lactating (b) and child-bearing age woman (c).

The disease was diagnosed based on the lab test results of the blood samples. The diagnosed results demonstrate that accompanying the rising of water iodine, the incidence of hypothyroxinemia and sub-clinical hypothyroidism among pregnant women, lactating women and women of child-bearing age were all increased (see Figure 3 a, b and c). When the odds ratio (OR) of morbidity was calculated for all pre-set water iodine levels using 50–99 µg/L as the control group, the result demonstrate that the OR of hypothyroxinemia among the pregnant women decreased across all groups but remained higher than the control group. The OR of sub-clinical hypothyroidism among lactating women gradually rose to eight times that of the control group, whereas the OR of sub-clinical hypothyroidism among the women of child-bearing age gradually rose to four times of that of the control group. The OR of clinical hyperthyroidism and hypothyroidism failed to be obtained due to too few cases (see Table 2).

**Table 2:** ORs for different pre-set water iodine levels 50-99 µg/L as the control group.

	Pre-set water	Hypo-	Sub-clinical	Clinical	Sub-clinical
Category	iodine groups	hyroxinemia	hypo-	hypo-	hyper-
	(µg/L)		thyroidism	thyroidism	thyroidism
Pregnant	100–149	1.82	0.00		1.05
	150–299	1.33	0.00		0.00
	≥300	1.13	1.12		0.00
Lactating	100–149	1.00	2.11	1.00	
women	150–299	0.00	3.35	0.00	
	≥300	0.47	8.14	1.00	
Other	100–149		0.47		
women	150–299		3.00		
	≥300		3.86		
Total	100∼149	1.48	0.59	2.07	1.01
	150∼299	1.00	1.69	2.03	1.00
	≥300	0.81	3.48	3.21	2.10

## Discussion

The goiter rate of Shanxi is excessively higher compared with other provinces with similar median water iodine levels. A possible contributor could be a different type of iodine within the water, such as iodate, iodine ion or other substances causing goiter in the environment. However, the specific contributor is subject to further study.

The study results can be fed into the refined definition elevated water iodine areas and endemic goiter areas. Children of all of the four provinces exhibited MUIs greater than 300 µg/L in the group with water iodine levels of 100–149 µg/L. Most of the children in the group with water iodine between 50 and 99 µg/L exhibited urinary iodine between 200 and 299 µg/L or more than adequate levels, whereas the MUI of pregnant women was 150–249 µg/L or adequate levels. When the water iodine samples were further broken down, the urinary iodine levels of the children demonstrate that the MUI of the children was 309.0 µg/L when the water iodine level was 90 to 100 µg/L and that the children's MUIs drop when the water iodine decreases. However, a substantial MUI decrease has not been registered among the children, with the average MUI of 241.6 µg/L being observed when the water iodine decreases to 50 to 60 µg/L. When the water iodine continues to drop, the MUI of the pregnant women also decreases to the lower bound of the adequate level at 150 µg/L. Therefore, it is safe and conservative to set the cut-off water iodine level at 100 µg/L in the new definition (see Table 3).

**Table 3:** MUI of the children under different water iodine levels.

Actual water iodine group(µg/L)	Urinary iodine of children		Urinary iodine of pregnant	
	Size	Median (µg/L)	Size	Median (µg/L)
290–299	18	551.2	2	350.5
280–289	18	576.7	14	259.9
270–279	28	510.1	7	299.4
260–269	127	468.0	11	329.9
250–259	270	411.8	52	338.7
240–249	135	482.0	70	367.5
230–239	122	360.6	54	289.6
220–229	26	443.6	43	318.2
210–219	23	523.3	13	370.7
200–209	14	339.0	16	338.8
190–199	15	514.0	15	335.4
180–189	14	300.0	14	235.0
170–179	114	277.8	13	172.0
160–169	30	397.8	19	298.4
150–159	27	254.3	29	254.3
140–149	347	344.1	124	301.4
130–139	37	363.9	21	209.0
120–129	80	346.3	46	216.4
110–119	372	339.0	93	306.4
100–109	108	332.7	30	268.7
90–99	120	309.0	16	241.8
80–89	195	285.0	39	235.6
70–79	237	259.9	33	153.2
60–69	289	256.4	57	165.9
50–59	169	241.6	30	183.9

The prevalence of sub-clinical hypothyroidism among women was 8.1% among the population above 18 years-old in Jiangsu. We determined that 8.2% of the survey subjects exhibited sub-clinical hypothyroidism in the group with water iodine levels of 50–99 µg/L, which is close to the survey result. The other research in Jiangsu was 18.9% and 11.1% for sub-clinical hypothyroidism and hypothyroidism in child-bearing age women[17]. In the screening of 4,800 pregnant women at eight-week gestation by the research team led by Prof. Teng WP, 6.15% of the subjects were determined to have sub-clinical hypothyroidism (9.5% for this project), 1.11% exhibited hypothyroxinemia (19.0% for this project), and 9.6% were TPO Ab positive (1.6% for this project). Sang ZN et al. found excessive iodine intake during late pregnancy may lead to maternal thyroid dysfunction, particularly subclinical hypothyroidism when their urinary above 250 µg/L[18]. The data suggest that a significant number of pregnant women exhibit “mild” thyroid dysfunction.

## Conclusions

The standard committee is revising the *Determination and classification of the areas of elevated water iodine and the endemic areas of iodine excess goiter* (GB/T19380-2003) according to the survey results. The new standard will upgrade the definition of areas of elevated water iodine (areas with water iodine exceeding 150 µg/L) to the areas with water iodine levels exceeding 100 µg/L. Furthermore, areas in which more than 5% of the local children between eight and 10 years-old exhibit goiter will be regarded as endemic areas. The new standard will be approved and released in the near future.

The *Surveillance Plan for the Areas with Elevated Water Iodine Levels in China (Trial)* has been developed, which determines that the surveillance will sample half of the elevated water iodine counties in Tianjin, Hebei, Shanxi, Jiangsu, Anhui, Shandong, Henan, and Shaanxi and that surveillance sites will be set up in the villages to survey water iodine levels, children's thyroid sizes, and children's urinary iodine. The plan will be revised and finalised within several years of trial implementation.

The survey results have indicated that the supply of non-iodised salt alone cannot ensure that the residents will have adequate iodine nutrition and that water improvement must be adopted as well. The need for water improvement is especially pressing for areas with water iodine levels exceeding 100 µg/L.

The goiter rate of the children in Shanxi is slightly higher than other provinces despite no significant differences in the water iodine levels. This observation suggests that other factors may be in play, such as a different type of iodine existing in the water or factors other than iodine.

With the increasing water iodine levels, the percentage of pregnant women suffering hypothyroxinemia rises before dropping, and the number of lactating women and women of child-bearing age with sub-clinical hypothyroidism increased eight-fold and four-fold, respectively. Additionally, the number of women who tested positive for TPO Ab increased, and the number of women who tested positive for TG Ab first increased then decreased, and those women positive for both TPO and TG Ab accounted for a high percentage.

It is reasonable to define areas with elevated water iodine as those with water iodine levels exceeding than 100 µg/L.

## Supporting Information

File S1 **This file contains Tables S1–S14 and Figures S1–S7.** Table S1, Project sites selected. Table S2, The sample size for the pre-set water iodine levels. Note: Please note that all the samples reflected in this and following tables are non-iodized salt users. Table S3, The sample size for the actual water iodine levels. Table S4, The number of blood samples collected according to the pre-set water iodine and the actual water iodine levels. Table S5, Age group distribution of the surveyed children. Table S6, Age group distribution of the surveyed women. Table S7, Actual household drinking water iodine levels of the children and pregnant women grouped as per the pre-set water iodine levels. Table S8, Urinary iodine levels of the children and pregnant women in the pre-set water iodine groups. Table S9, Urinary iodine frequency distribution of the children in the pre-set water iodine groups. Table S10, Urinary iodine frequency distribution of the children in the actual water iodine groups. Table S11, Urinary iodine frequency distribution of the pregnant women in the pre-set water iodine groups. Table S12, Urinary iodine frequency distribution of the pregnant women grouped in the actual water iodine groups. Table S13, Goiter rate of the children in the pre-set water iodine groups. Table S14, Results of the blood tests among the women in the pre-set water iodine groups. Note: The reference range is the standard range provided by the testing method and applies to the pregnant, lactating and general women. No specific reference range has been provided for different groups of subjects. Figure S1, Water iodine levels of the children and women grouped by the pre-set water iodine levels. Figure S2, Water iodine levels of the children and pregnant women in the actual water iodine groups. Figure S3, MUI of the children in the pre-set and actual water iodine groups. Figure S4, MUI of the pregnant women in the pre-set and actual water iodine groups. Figure S5, Relations between median water iodine and MUI of the subjects in the pre-set water iodine groups. Figure S6, Goiter rate of the children in the pre-set water iodine groups Correlation between children's water iodine and urinary iodine. Figure S7, Goiter rate of the children in the actual water iodine groups.
(DOC)
