# Supplementary material for: The Standard, Intervention Measures and Health Risk for High Water Iodine Areas
Source: PLoS One. 2014 Feb 28;9(2):e89608. doi: 10.1371/journal.pone.0089608 (PMC3938487; doi:10.1371/journal.pone.0089608)
Supplement: File S1 — This file contains Tables S1–S14 and Figures S1–S7. Table S1, Project sites selected. Table S2, The sample size for the pre-set water iodine levels. Note: Please note that all the samples reflected in this and following tables are non-iodized salt users. Table S3, The sample size for the actual water iodine levels. Table S4, The number of blood samples collected according to the pre-set water iodine and the actual water iodine levels. Table S5, Age group distribution of the surveyed children. Table S6, Age group distribution of the surveyed women. Table S7, Actual household drinking water iodine levels of the children and pregnant women grouped as per the pre-set water iodine levels. Table S8, Urinary iodine levels of the children and pregnant women in the pre-set water iodine groups. Table S9, Urinary iodine frequency distribution of the children in the pre-set water iodine groups. Table S10, Urinary iodine frequency distribution of the children in the actual water iodine groups. Table S11, Urinary iodine frequency distribution of the pregnant women in the pre-set water iodine groups. Table S12, Urinary iodine frequency distribution of the pregnant women grouped in the actual water iodine groups. Table S13, Goiter rate of the children in the pre-set water iodine groups. Table S14, Results of the blood tests among the women in the pre-set water iodine groups. Note: The reference range is the standard range provided by the testing method and applies to the pregnant, lactating and general women. No specific reference range has been provided for different groups of subjects. Figure S1, Water iodine levels of the children and women grouped by the pre-set water iodine levels. Figure S2, Water iodine levels of the children and pregnant women in the actual water iodine groups. Figure S3, MUI of the children in the pre-set and actual water iodine groups. Figure S4, MUI of the pregnant women in the pre-set and actual water iodine groups. Figure S5, Relations between median w [file pone.0089608.s001.doc]

Supporting Information file 
Table S1  Project sites selected 
Provinces	Water iodine£¨μg/L£©	Project sites   county£¨Township£©	
			
Shandong	50¡«99	Xinxian(Zudian, Gucheng, Shibalipu), Guanxian (Xinji, ingshui), Chiping£¨Fengtun, Caitun£©	
	100¡«149	Xinxian(Zudian, Gucheng, Shibalipu), Guanxian (Xinji, Qingshui) and Chiping County£¨Fengtun, Caitun£©	
	150¡«299	Xinxian(Gucheng), Guanxian(Xinji, Qingshui) and Chiping Fengtun, Caitun£©	
	≥300	Xinxian(Gucheng, Shibalipu), Guanxian(Xinji, Qingshui) and Chiping£¨Fengtun, Caitun£©	
Shanxi	50¡«99	Xiaodian(Xiwenzhuang, Beige), Qingxu(Wangda)	
	100¡«149	Xiaodian(Beige)	
	150¡«299	Qingxu(Wangda, Xigu)	
	≥300	Xiaodian(Xiwenzhuang), Qingxu (Mengfeng£©	
Henan	50¡«99	Xiayi(Zhongfeng), Taiqian (Dayuchen)	
	100¡«149	Xiayi (Caoji), Taiqian (Qingshuihe), Yucheng (Zhangji)	
	150¡«299	Xiayi (Yangji), Taiqian (Houmiao), Yucheng (Zhangji)	
	≥300	Xiayi (Yangji), Taiqian (Dayuchen), Yucheng (Zhangji)	
Hebei	100¡«149	Weixian (Zhaocun)	
	150¡«299	Dacheng city (Zangtun)	
	≥300	Huanghua city (Yangerzhuang)	


Table S2 The sample size for the pre-set water iodine levels
Province	Pre-set Water iodine group
	Children	Pregnant	
		Planned sample size for Water, 	Actual sample size(for non-iodized salt users)	Planned sample size for Water, 	Actual sample size(for non-iodized salt users)	
	(μg/L)	Urinary, Salt	Water	Urine	Salt	Urinary, Salt	Water	Urine	Salt	
Shandong	50¡«99	600	684	684	684	150-300	144	144	144	
	100¡«149	200	254	254	254	50-100	85	85	85	
	150¡«299	200	306	306	306	50-100	62	62	62	
	≥300	200	214	214	214	50-100	78	80	80	
Shanxi	50¡«99	200	196	196	196	50-100	21	20	21	
	100¡«149	200	189	189	189	50-100	20	18	20	
	150¡«299	200	159	158	159	50-100	21	21	21	
	≥300	200	165	165	165	50-100	19	19	19	
Henan	50¡«99	0	200	198	200	0	65	65	65	
	100¡«149	200	354	352	354	50-100	158	158	158	
	150¡«299	200	314	314	314	50-100	156	156	156	
	≥300	200	306	304	306	50-100	151	151	151	
Hebei	100¡«149	200	195	195	195	50-100	50	50	50	
	150¡«299	200	226	213	226	50-100	50	50	50	
	≥300	200	211	211	211	50-100	54	54	54	
Total	50¡«99	800	1080	1078	1080	200-400	230	229	230	
	100¡«149	800	992	990	992	200-400	313	311	313	
	150¡«299	800	1005	991	1005	200-400	289	289	289	
	≥300	800	896	894	896	200-400	302	304	304	
Note£ºPlease note that all the samples reflected in this and following tables are non-iodized salt users.

Table S3 The sample size for the actual water iodine levels
Province	Actual Water 	Children	Pregnant	
	iodine group  	Planned sample size for Water, 	Actual sample size(for non-iodized salt users)	Planned sample size for Water, 	Actual sample size(for non-iodized salt users)	
	(μg/L)	Urinary, Salt	Water	Urine	Salt	Urinary, Salt	Water	Urine	Salt	
Shandong	50¡«99	600	683	683	683	150-300	142	142	142	
	100¡«149	200	256	256	256	50-100	85	85	85	
	150¡«299	200	302	302	302	50-100	62	62	62	
	≥300	200	215	215	215	50-100	79	79	79	
Shanxi	50¡«99	200	196	196	196	50-100	21	20	21	
	100¡«149	200	189	189	189	50-100	20	18	20	
	150¡«299	200	159	158	159	50-100	21	21	21	
	≥300	200	165	165	165	50-100	19	19	19	
Henan	50¡«99	0	131	129	131	0	26	26	26	
	100¡«149	200	338	336	338	50-100	147	147	147	
	150¡«299	200	383	383	383	50-100	205	205	205	
	≥300	200	310	308	310	50-100	150	150	150	
Hebei	100¡«149	200	195	195	195	50-100	50	50	50	
	150¡«299	200	226	213	226	50-100	65	65	65	
	≥300	200	211	211	211	50-100	39	39	39	
Total	50¡«99	800	1010	1008	1010	200-400	187	186	187	
	100¡«149	800	978	976	978	200-400	299	297	299	
	150¡«299	800	1070	1056	1070	200-400	353	353	353	
	≥300	800	901	899	901	200-400	287	287	287	


Table S4 The number of blood samples collected according to the pre-set water iodine and the actual water iodine levels
Province	Water iodine	Pregnant/Lactate/Reproductive age women blood sample	
	£¨μg/L£©	Planned sample size	Actual sample size	
Shanxi	50¡«99	21/20/20	21/20/20	
	100¡«149	20/20/20	20/20/20	
	150¡«299	21/20/20	21/20/20	
	≥300	19/20/20	19/20/20	


Table S5  Age group distribution of the surveyed children
Province	Pre-set water iodine group (μg/L£©	Age 6	Age 7	Age 8	Age 9	Age 10	Age 11	Age 12	
Shandong	50¡«99	12.4	18.7	15.1	19.4	13.5	14.6	6.3	
	100¡«149	13.0	15.0	11.4	13.4	14.6	22.8	9.8	
	150¡«299	0.7	16.3	21.6	20.3	13.4	18.6	9.2	
	≥300	9.3	14.0	22.9	23.4	13.6	12.6	4.2	
Shanxi	50¡«99	1.0	8.2	14.3	19.9	33.7	17.3	5.1	
	100¡«149	0	0	16.9	30.2	22.2	18.5	9.0	
	150¡«299	0	0	13.2	33.3	40.3	12.6	0.6	
	≥300	4.2	12.1	9.7	22.4	17.0	18.2	15.2	
Henan	50¡«99	11.5	6.0	15.5	40.5	17.5	5.5	3.5	
	100¡«149	4.0	12.7	24.6	20.9	19.5	12.1	6.2	
	150¡«299	0	6.1	21.0	31.8	32.8	7.6	0.6	
	≥300	0	4.9	28.4	30.4	29.4	4.9	2.0	
Hebei	100¡«149	0	7.2	21.5	34.9	33.8	2.6	0	
	150¡«299	0	21.7	23.9	13.3	19.9	21.2	0	
	≥300	0	0	16.6	54.0	29.4	0	0	
Total	50¡«99	10.2	14.4	15.0	23.4	17.9	13.4	5.6	
	100¡«149	4.7	9.8	19.2	23.5	21.6	14.2	6.5	
	150¡«299	0.2	11.7	20.6	24.4	25.2	14.8	3.1	
	≥300	3.0	7.3	20.9	32.8	23.3	8.0	4.5	


Table S6  Age group distribution of the surveyed women
Province	Pre-set water iodine group£¨μg/L£©	20-	25-	30-	35-	40-	
Shandong	50¡«99	33.3	25.7	34.7	2.8	3.5	
	100¡«149	28.2	32.9	29.4	9.4	0	
	150¡«299	41.0	32.8	21.3	1.6	3.3	
	≥300	53.8	20.0	25.0	1.3	0	
Shanxi	50¡«99	19.0	33.3	28.6	19.0	0.0	
	100¡«149	55.0	40.0	5.0	0.0	0.0	
	150¡«299	23.8	66.7	9.5	0.0	0.0	
	≥300	47.4	36.8	10.5	5.3	0.0	
Henan	50¡«99	55.4	30.8	7.7	3.1	1.5	
	100¡«149	45.6	43.7	8.9	1.9	0	
	150¡«299	58.3	32.7	6.4	0.6	0	
	≥300	51.0	35.8	9.3	2.6	1.3	
Hebei	100¡«149	24.0	52.0	20.0	4.0	0	
	150¡«299	58.0	36.0	6.0	0	0	
	≥300	63.0	25.9	11.0	0	0	
Total	50¡«99	38.4	27.9	26.6	4.4	2.6	
	100¡«149	38.0	41.9	16.0	4.2	0.0	
	150¡«299	52.1	35.8	9.7	0.7	1.7	
	≥300	53.6	29.9	13.8	2.0	0.7	


Table S7 Actual household drinking water iodine levels of the children and pregnant women grouped as per the pre-set water iodine levels
Province	Pre-set water iodine 	Household drinking water of children 	Household drinking water of pregnant	
	group(μg/L)	size	Median of water iodine(μg/L)	size	Median of water iodine(μg/L)	
Shandong	50¡«99	684	71.6	144	68.3	
	100¡«149	254	113.8	85	120.2	
	150¡«299	306	239.2	62	201.6	
	≥300	214	350.6	78	405.4	
Shanxi	50¡«99	196	73.8	21	82	
	100¡«149	189	144.7	20	121.5	
	150¡«299	159	258.5	21	258.5	
	≥300	165	501.0	19	485.9	
Henan	50¡«99	200	64.1	65	135.3	
	100¡«149	354	148.3	158	148.0	
	150¡«299	314	242.8	156	237.9	
	≥300	306	475.7	151	475.7	
Hebei	100¡«149	195	111.7	50	140.7	
	150¡«299	226	236.5	50	233.1	
	≥300	211	392.3	54	335.8	
Total	50¡«99	1080	73.8	230	73.8	
	100¡«149	992	118.8	313	121.5	
	150¡«299	1005	242.8	289	289.0	
	≥300	896	392.6	302	399.6	

Table S8  Urinary iodine levels of the children and pregnant women in the pre-set water iodine groups
Province	Pre-set water iodine group	Children	Pregnant	
	(μg/L)	Size	Median of urinary iodine (μg/L)	Size	Median of urinary iodine (μg/L)	
Shandong	50¡«99	684	253.0	144	166.9	
	100¡«149	254	338.0	85	202.9	
	150¡«299	306	457.3	62	278.8	
	≥300	214	621.0	80	469.0	
Shanxi	50¡«99	196	274.2	20	238.6	
	100¡«149	189	312.8	18	204.9	
	150¡«299	158	445.6	21	373.9	
	≥300	165	793.5	19	607.4	
Henan	50¡«99	198	279.0	65	247.8	
	100¡«149	352	340.4	158	318.0	
	150¡«299	314	383.7	156	317.85	
	≥300	304	346.0	151	351.0	
Hebei	100¡«149	195	396.4	50	309.8	
	150¡«299	213	316.8	50	256.8	
	≥300	211	382.7	54	386.20	
Total	50¡«99	1078	262.7	229	192.1	
	100¡«149	990	339.0	311	282.9	
	150¡«299	991	402.2	289	309.2	
	≥300	894	474.2	304	389.0	


Table S9 Urinary iodine frequency distribution of the children in the pre-set water iodine groups
Province	Pre-set water 	Frequency distribution of children urinary iodine (%)	
	iodine group  (μg/L)	Size	<50	50-100	100-150	150-200	200-300	300-500	≥500	
Shandong	50¡«99	684	1.0	6.9	10.4	14.3	28.5	27.9	11.0	
	100¡«149	254	0.4	1.6	7.9	9.8	21.3	40.6	18.5	
	150¡«299	306	1.6	1.0	4.2	6.9	10.1	31.0	45.1	
	≥300	214	0	1.4	0.9	3.7	5.6	22.9	65.4	
Shanxi	50¡«99	196	0	1.0	4.1	4.6	43.9	43.9	2.6	
	100¡«149	189	0	0	0	0	43.4	42.3	14.3	
	150¡«299	158	0	0	0	0	5.7	60.1	34.2	
	≥300	165	0	0	0.6	0	1.8	9.7	87.9	
Henan	50¡«99	198	0.5	2.5	9.1	7.1	35.4	35.9	9.6	
	100¡«149	352	0	0.9	5.7	9.4	24.4	34.1	25.6	
	150¡«299	314	0	0.6	2.9	7.3	23.2	35.4	30.6	
	≥300	304	0	1.6	4.3	7.2	24.0	35.2	27.6	
Hebei	100¡«149	195	0	1.5	5.6	6.7	18.5	39.0	28.7	
	150¡«299	213	0	4.2	4.7	9.9	25.8	39.9	15.5	
	≥300	211	4.3	0.5	3.8	6.2	16.6	52.1	16.6	
Total	50¡«99	1078	0.7	5.0	9.0	11.2	32.6	32.3	9.2	
	100¡«149	990	0.1	1.0	5.2	7.2	26.1	38.3	22.2	
	150¡«299	991	0.5	1.4	3.2	6.6	17.0	39.0	32.4	
	≥300	894	1.0	1.0	2.7	4.8	13.8	31.5	45.2	


Table S10 Urinary iodine frequency distribution of the children in the actual water iodine groups
Province	Actual water 	Frequency distribution of children urinary iodine (%)	
	Iodine groups  (μg/L)	Size	<50	50-100	100-150	150-200	200-300	300-500	≥500	
Shandong	50¡«99	683	1.0	6.9	10.4	14.5	28.6	27.7	11.0	
	100¡«149	256	0.4	1.6	7.8	9.8	21.5	40.6	18.4	
	150¡«299	302	1.7	1.0	4.3	6.6	9.9	30.8	45.7	
	≥300	215	0.0	1.4	0.9	3.7	5.6	23.3	65.1	
Shanxi	50¡«99	196	0.0	1.0	4.1	4.6	43.9	43.9	2.6	
	100¡«149	189	0.0	0.0	0.0	0.0	43.4	42.3	14.3	
	150¡«299	158	0.0	0.0	0.0	0.0	5.7	60.1	34.2	
	≥300	165	0.0	0.0	0.6	0.0	1.8	9.7	87.9	
Henan	50¡«99	129	0.0	0.0	4.7	9.3	46.5	36.4	3.1	
	100¡«149	336	0.3	1.8	6.3	8.9	24.7	32.1	25.9	
	150¡«299	383	0.0	0.8	4.2	6.8	21.9	37.3	29.0	
	≥300	308	0.0	1.6	4.9	7.5	23.7	34.7	27.6	
Hebei	100¡«149	195	0.0	1.5	5.6	6.7	18.5	39.0	28.7	
	150¡«299	213	0.0	4.2	4.7	9.9	25.8	39.9	15.5	
	≥300	211	4.3	0.5	3.8	6.2	16.6	52.1	16.6	
Total	50¡«99	1008	0.7	4.9	8.4	11.9	33.8	31.9	8.3	
	100¡«149	976	0.2	1.3	5.3	7.0	26.2	37.7	22.2	
	150¡«299	1056	0.5	1.4	3.7	6.3	16.9	39.4	31.8	
	≥300	899	1.0	1.0	2.9	4.9	13.7	31.5	45.1	


Table S11 Urinary iodine frequency distribution of the pregnant women in the pre-set water iodine groups
Province	Pre-set water iodine groups	Frequency distribution of pregnant urinary iodine (%)	
	(μg/L)	Size	<50	50-100	100-150	150-250	250-300	300-500	≥500	
Shandong	50¡«99	144	2.1	18.1	22.2	30.6	6.2	16.7	4.2	
	100¡«149	85	1.2	9.4	23.5	27.1	19.9	17.6	1.2	
	150¡«299	62	0	3.2	16.1	25.8	14.5	25.8	14.5	
	≥300	80	0	2.5	1.3	11.0	4.2	33.8	47.5	
Shanxi	50¡«99	20	0.0	5.0	20.0	30.0	15.0	30.0	0.0	
	100¡«149	18	0.0	5.6	0.0	66.7	5.6	16.7	5.6	
	150¡«299	21	0.0	0.0	0.0	14.3	4.8	52.4	28.6	
	≥300	19	0	0	0	0	10.53	21.0	68.4	
Henan	50¡«99	65	3.1	4.6	16.9	26.2	6.1	27.7	15.4	
	100¡«149	158	1.3	1.9	5.1	18.4	13.2	41.1	19.0	
	150¡«299	156	0	0.6	10.3	17.9	13.5	42.3	15.4	
	≥300	151	0.7	1.3	4.6	15.9	9.9	43.7	23.8	
Hebei	100¡«149	50	32.0	0.0	0.0	4.0	8.0	24.0	32.0	
	150¡«299	50	0.0	12.0	8.0	28.0	14.0	28.0	10.0	
	≥300	54	1.9	3.7	1.9	16.7	7.4	55.6	13.0	
Total	50¡«99	229	2.2	13.0	20.4	29.1	7.0	20.9	7.0	
	100¡«149	311	6.0	3.8	8.8	22.3	13.5	29.9	15.1	
	150¡«299	289	0.0	3.1	10.4	21.1	13.1	37.0	15.2	
	≥300	304	0.6	2.0	3.0	13.8	7.9	41.8	30.9	


Table S12 Urinary iodine frequency distribution of the pregnant women grouped in the actual water iodine groups
Province	Actual water iodine groups 	Frequency distribution of pregnant urinary iodine (%)	
	(μg/L)	Size	<50	50-100	100-150	150-250	250-300	300-500	≥500	
Shandong	50¡«99	142	2.1	18.3	22.5	30.3	6.3	16.9	3.5	
	100¡«149	85	1.2	9.4	23.5	27.0	20.0	17.6	1.2	
	150¡«299	62	0.0	3.2	16.1	17.2	23.1	25.8	14.5	
	≥300	79	0.0	2.5	1.3	3.8	12.7	32.9	46.8	
Shanxi	50¡«99	20	0.0	5.0	20.0	30.0	15.0	30.0	0.0	
	100¡«149	18	0.0	5.6	0.0	66.7	5.6	16.7	5.6	
	150¡«299	21	0.0	0.0	0.0	14.3	4.8	52.4	28.6	
	≥300	19	0.0	0.0	0.0	0.0	10.5	21.1	68.4	
Henan	50¡«99	26	3.8	3.8	7.7	30.8	2.8	42.3	7.7	
	100¡«149	147	0.0	2.7	8.2	14.3	12.2	42.2	20.4	
	150¡«299	205	1.0	1.0	10.2	21.0	13.6	37.6	15.6	
	≥300	150	0.7	1.3	4.7	17.3	9.3	42.7	24.0	
Hebei	100¡«149	50	32.0	0.0	0.0	4.0	8.0	24.0	32.0	
	150¡«299	65	0.0	9.2	6.2	27.7	13.9	35.4	7.7	
	≥300	39	2.6	5.1	2.6	12.8	5.2	53.8	17.9	
Total	50¡«99	186	2.2	15.1	20.4	30.6	7.0	22.0	3.8	
	100¡«149	297	5.7	4.4	10.8	19.5	13.5	31.0	16.2	
	150¡«299	353	0.6	2.8	9.9	22.7	13.3	36.0	14.7	
	≥300	287	0.7	2.1	3.1	14.3	7.3	40.1	32.4	


Table S13 Goiter rate of the children in the pre-set water iodine groups
Province	Pre-set water iodine groups	Children	
	(μg/L)	Size	Goiter rate (%)	
Shandong	50¡«99	684	0.6	
	100¡«149	254	1.6	
	150¡«299	306	4.6	
	≥300	214	7.0	
Shanxi	50¡«99	196	5.6	
	100¡«149	189	13.2	
	150¡«299	159	12.6	
	≥300	165	10.9	
Henan	50¡«99	200	4.0	
	100¡«149	354	2.8	
	150¡«299	314	1.3	
	≥300	306	4.2	
Hebei	100¡«149	195	5.1	
	150¡«299	226	4.4	
	≥300	211	3.8	
Total	50¡«99	1078	2.2	
	100¡«149	990	5.1	
	150¡«299	1005	5.0	
	≥300	895	6.3	

Table S14 Results of the blood tests among the women in the pre-set water iodine groups.
Category	Pre-set water iodine groups 	T3(2.8-7.1)	T4(12-22)	TSH(0.27-4.20)	TgAb 	TPOAb 	Rate of positive with either 	
	(μg/L)	L	H	L	H	L	H	(>115)	(>34)	anti-TP or anti-Tg	
Pregnant	50¡«99	0.0	0.0	19.0	0.0	4.8	9.5	9.5	0.0	0.0	
	100¡«149	0.0	0.0	30.0	0.0	5.0	0.0	5.0	0.0	0.0	
	150¡«299	4.8	0.0	33.3	0.0	0.0	9.5	0.0	4.8	0.0	
	≥300	10.5	0.0	31.6	0.0	0.0	21.1	0.0	0.0	0.0	
Lactating	50¡«99	0.0	0.0	10.0	0.0	0.0	10.0	0.0	0.0	0.0	
women	100¡«149	0.0	5.0	10.0	5.0	5.0	15.0	15.0	30.0	10.0	
	150¡«299	0.0	0.0	0.0	0.0	5.0	15.0	20.0	10.0	10.0	
	≥300	0.0	0.0	5.0	0.0	10.0	35.0	20.0	10.0	10.0	
General	50¡«99	0.0	0.0	0.0	0.0	0.0	10.0	10.0	5.0	5.0	
women	100¡«149	0.0	5.0	5.0	5.0	5.0	10.0	10.0	20.0	5.0	
	150¡«299	0.0	0.0	0.0	0.0	0.0	25.0	15.0	20.0	10.0	
	≥300	0.0	0.0	0.0	0.0	0.0	30.0	15.0	5.0	5.0	
Total	50¡«99	0.0	0.0	9.8	0.0	1.6	9.8	6.6	1.6	1.6	
	100¡«149	0.0	3.3	15.0	3.3	5.0	8.3	10.0	16.7	5.0	
	150¡«299	1.6	0.0	11.5	0.0	1.6	16.4	11.5	11.5	6.6	
	≥300	3.4	0.0	11.9	0.0	3.4	28.8	11.9	5.1	5.1	
Note: The reference range is the standard range provided by the testing method and applies to the pregnant, lactating and general women. No specific reference range has been provided for different groups of subjects.


Figure S1 Water iodine levels of the children and women grouped by the pre-set water iodine levels


Figure S2  Water iodine levels of the children and pregnant women in the actual water iodine groups


Figure S3  MUI of the children in the pre-set and actual water iodine groups


Figure S4  MUI of the pregnant women in the pre-set and actual water iodine groups 


Figure S5  Relations between median water iodine and MUI of the subjects in the pre-set water iodine groups


Figure S6  Goiter rate of the children in the pre-set water iodine groups
Correlation between children’s water iodine and urinary iodine


Figure S7 Goiter rate of the children in the actual water iodine groups
